# *Cis*-regulatory variations: A study of SNPs around genes showing *cis*-linkage in segregating mouse populations

**DOI:** 10.1186/1471-2164-7-235

**Published:** 2006-09-15

**Authors:** Debraj GuhaThakurta, Tao Xie, Manish Anand, Stephen W Edwards, Guoya Li, Susanna S Wang, Eric E Schadt

**Affiliations:** 1 Genetics, Rosetta Inpharmatics LLC, a wholly owned subsidiaryof Merck & Co., Inc. 401 Terry Avenue North, Seattle, WA 98109, USA; 2 Informatics, Rosetta Inpharmatics LLC, a wholly owned subsidiary of Merck & Co., Inc. 401 Terry Avenue North, Seattle, WA 98109, USA; 3 Department of Medicine, David Geffen School of Medicine, University of California, Los Angeles, CA 90095-1679, USA; 4 Microsoft Corporation, One Microsoft Way, Redmond, WA 98052-6399, USA

## Abstract

**Background:**

Changes in gene expression are known to be responsible for phenotypic variation and susceptibility to diseases. Identification and annotation of the genomic sequence variants that cause gene expression changes is therefore likely to lead to a better understanding of the cause of disease at the molecular level. In this study we investigate the pattern of single nucleotide polymorphisms (SNPs) in genes for which the mRNA levels show *cis*-genetic linkage (gene expression quantitative trait loci mapping in *cis*, or *cis*-eQTLs) in segregating mouse populations. Such genes are expected to have polymorphisms near their physical location (*cis*-variations) that affect their mRNA levels by altering one or more of the *cis*-regulatory elements. This led us to characterize the SNPs in promoter (5 Kb upstream) and non-coding gene regions (introns and 5 Kb downstream) (*cis*-SNPs) and the effects they may have on putative transcription factor binding sites.

**Results:**

We demonstrate that the *cis*-eQTL genes (CEGs) have a significantly higher frequency of *cis*-SNPs compared to non-CEGs (when both sets are taken from the non-IBD regions, i.e. regions not identical by descent). Most CEGs having *cis*-SNPs do not contain these SNPs in the phylogenetically conserved regions. In those CEGs that contain *cis*-SNPs in the phylogenetically conserved regions, enrichment of *cis*-SNPs occurs both within and outside of the conserved sequences. A higher fraction of CEGs are also seen to harbor *cis*-SNP that affect predicted transcription factor binding sites, a likely consequence of the higher *cis*-SNPs density in these genes.

**Conclusion:**

This present study provides the first genome-wide investigation of the putative *cis*-regulatory variations in a large set of genes whose levels of expression give rise to *cis*-linkage in segregating mammalian populations. Our results provide insights into the challenges that exist in identifying polymorphisms regulating gene expression using bioinformatic sequence analysis approaches. The data provided herein should benefit future investigations in this area.

## Background

Single nucleotide polymorphisms (SNPs) in the genomic sequence underlie susceptibility to or protection from diseases by affecting biological processes at the molecular level, such as protein structure, transcription, alternative splicing etc [1]. There are a number of examples in which polymorphisms in the promoter regions, and those causing expression changes in the corresponding genes, have been found to be associated with disease [2-5]. In addition, genetic variation of gene expression has been utilized to identify causal genes for complex diseases [6-8]. However, the pattern of polymorphisms that underlie heritable variation of gene expression in segregating mammalian populations, as well as bioinformatic sequence analysis methods for identifying these regulatory polymorphisms, have not yet been investigated in a systematic way. Here we characterize the pattern of *cis*-SNPs that could cause quantitative genetic variations in mRNA levels in two mouse intercross populations.

We investigated the frequency and the potential role of the *cis*-SNPs for disrupting transcription factor binding sites (TFBS) around the genes whose expression levels in murine intercross populations gave rise to strong *cis*-acting eQTL. We focused on this set of genes for the following reasons: 1) a sizable fraction of genes whose expression varies in a segregating population show *cis*-linkage [9-12], 2) evidence for the medical importance of *cis*-regulatory variation has been demonstrated by positional cloning studies in which SNPs in susceptibility genes that were not located in the protein coding or splice-site regions were nevertheless shown to be associated with complex human diseases such as stroke, type 2 diabetes etc. [4,13,14], 3) the polymorphisms that affect the expression levels of these genes are either in the genomic region of the gene or in the nearby upstream or downstream region (*cis*-regulatory variation [4]), which significantly restricts the search space for these causal variations.

We found a significantly higher number of *cis*-acting eQTL genes (*CEGs*) were in regions that were not identical by descent (IBD) between the parental inbred mouse lines used to construct the mouse crosses. In considering the genes that fall outside of these IBD regions, we found that a significantly higher number of CEGs have *cis*-SNPs in their promoter (i.e. immediate 5' upstream sequence) and non-coding regions (i.e. introns and immediate 3' downstream sequences) compared to genes that do not give rise to *cis*-acting eQTLs (non-CEGs). The density of SNPs in these regions is also significantly higher in the CEGs compared to non-CEGs. In addition, the enrichment of *cis*-SNPs is not limited to the highly conserved sequences between mouse and human, and in fact in a majority of the CEGs the *cis*-SNPs do not overlap any conserved sequences in the promoter or non-coding regions, suggesting that the *cis*-SNPs in these genes do not perturb the highly conserved sequences in the immediate vicinity. A higher fraction of CEGs have *cis*-SNPs that perturb predicted transcription factor binding sites (TFBS) in non-coding regions, likely a consequence of the higher *cis*-SNP density in these regions resulting in an increased number of intersections between *cis*-SNPs and the TFBSs.

The implications of the above findings on the challenges related to the identification and annotation of genomic regulatory polymorphisms through bioinformatic sequence analysis methods are discussed. Our results suggest that the approaches that are commonly employed in identification of putative regulatory variants, such as searches for polymorphisms in the immediate upstream regions and cross-species conserved sequences, are unlikely to elucidate a significant fraction of the *cis*-regulatory variations responsible for causing changes in gene expression in genetically segregating mammalian populations.

## Results

### Mouse intercross populations and cis-acting eQTL genes

mRNA expression data for multiple tissues in F_2 _animals from two mouse intercrosses constructed from C57BL/6J and DBA/2J (referred here as the BXD cross) [10,15], and from C57BL/6J and C3H/HeJ inbred lines (referred here as the BXH cross) [16], were available for analysis (for details see methods). The BXD F_2 _population [10,15] consisted of 111 female mice and comprehensive mRNA expression profiles were available for liver, while the BXH F_2 _population [16] contained 334 mice (169 female, 165 male) and expression profiles were available for four tissues, namely liver, white adipose, whole brain, and skeletal muscle. All of the expression data from the two crosses we have used here for analyses were generated and described previously [6,10,16].

In the same manner as classic phenotypic trait data, QTLs for gene expression levels can be computationally mapped using genetic linkage mapping strategies [10]. mRNA levels of genes were treated as continuous variables and mapped to the genome using a standard interval mapping procedure [17] to identify expression QTLs (eQTLs). From the linkage results CEGs were defined as follows: 1) eQTL LOD score ≥ 4.3 (the threshold in an F_2 _mouse intercross for achieving a genome-wide p-value of 0.05, and a point-wise significance of 0.00005), 2) eQTL is near the physical location of the gene itself (within 10 Mb, equivalent to roughly 5 cM), 3) the eQTL explains more than 10% of the genetic variation of expression for the gene in the respective F_2 _populations.

Using the specific conditions described above, a total of 3,769 distinct CEGs were identified (roughly 20% of the genes represented on the array) over all four tissues in the BXH cross, and 338 CEGs were identified in BXD cross. Reasons for identification of significantly fewer CEGs in the BXD cross relative to the BXH cross include: 1) availability of mRNA profiles from only one (liver) tissue compared to four tissues in the BXH cross, and 2) a lower number of animals (111 in BXD compared to 334 in BXH) resulting in a reduced power to detect QTLs. The number of CEGs for the BXD cross given here is less than previously reported for this same cross [10,11], given we employed a more conservative definition of *cis*-eQTLs in this preset study for the purpose of minimizing the false positive calls and working with the highest confidence CEGs. The CEGs from all tissues in both crosses are provided in the supplementary materials (Additional files 1 and 2). Later we have described how we have prepared a common set of CEGs and non-CEGs for analysis by combining the data from the two crosses.

### IBD regions between parental strains and the distribution of CEGs with respect to the IBD status

Genomic segments in different mouse strains that are inherited from a common ancestor are referred to as identical by descent or IBD. The IBD regions can be considered to be largely homologous sequence blocks between two strains, while the non-IBD (nIBD) regions can be considered as polymorphic blocks. Most of the polymorphisms between mouse strains exist in sequence regions that are not in IBD [18], and reported variations that are in the IBD regions either represent sequencing errors or mutations that occurred in the strains after sub-speciation.

So that the readers can focus on the key findings of the manuscript, we present the details of the IBD map we have used here [19] and the reason for its selection in the Data and Methods section. However, it is worth mentioning here briefly that a very significant enrichment of CEGs was observed in the regions that were not in IBD (nIBD) (with the Fisher exact test p = 8.87 × 10^-28 ^for CEGs from the BxD cross, and 4.08 × 10^-296 ^for CEGs from the BxH cross). The analyses described in subsequent sections were performed with the set of genes that are *not *in IBD regions. This is because genes in IBD regions would be expected to have significantly fewer SNPs if any, in the surrounding regions, and therefore the comparison of patterns of polymorphisms in those genes with the CEGs, most of which contain *cis*-SNPs and are in nIBD, would not be appropriate.

**Figure 1:**
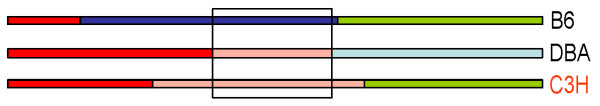
**Determining nIBD-BXH regions from IBD blocks between mouse strains**. B6 refers to C57BL/6J, DBA refers to DBA/2J, and C3H refers to C3H/HeJ. Horizontal bars represent genomic sequence. Regions that are in the same color between two or more strains represent the IBD blocks between those strains. nIBD-BXH (indicated with a box) are regions that are IBD between C3H/HeJ and DBA/2J, but nIBD between C3H/HeJ and C57BL/6J, and nIBD between C57BL/6J and DBA/2J, as explained in the text.

Since the C3H strain has not yet been sequenced, a complete set of SNPs between the B6 and C3H parental strains used to construct the BXH cross was not available from public sources or the Celera mouse SNP database [20]. Only a small number of SNPs (25,064) that mapped uniquely to the mouse genome were available between these two strains (from the public dbSNP database [21] (build 120)). Therefore, we used the set of SNPs that were polymorphic between B6 and DBA for analysis of the *cis*-SNPs around CEGs in the BXH cross as described below, imputing the regions of shared haplotypes between strains using the IBD map. Genomic sequence blocks that were IBD between C3H and DBA, but nIBD between B6 and C3H and nIBD between B6 and DBA (see Figure 1), were identified. These regions are called nIBD-BXH for reference. The nIBD-BXH regions identified in this way are expected to be homologous between C3H and DBA, but polymorphic between B6 and DBA as well as between B6 and C3H. In the nIBD-BXH regions, SNPs occurring between B6 and DBA should thus be the same as those occurring between B6 and C3H. Of the 492,250 SNPs polymorphic between B6 and DBA identified as falling in non-repeat regions, 274,908 (55.4%) were nIBD-BXH. Genes and SNPs contained in the nIBD-BXH regions were used for analysis of data from the BXH cross.

### Creating a common set of CEGs and non-CEGs from the BXD and BXH crosses

To characterize the frequency and location of *cis*-SNPs in genes, we constructed a common set of CEGs and non-CEGs from the BXD and BXH crosses. Given the number of CEGs identified in the BXD cross was small (~10% of the total available from both crosses), these data on their own would not be as highly powered to identify *cis*-SNP patterns of interest in the CEGs. Therefore, we combined the CEGs from the BXD and BXH crosses to carry out all subsequent analyses. Combining the 338 CEGs from BXD cross with the 3,769 CEGs from the BXH cross, and only considering the CEGs within the nIBD-BXH regions, resulted in a set of 2,047 distinct CEGs (see Additional file 2). The inclusion CEGs from the BXD cross added 75 distinct CEGs into the BXH data set. For the purpose of comparison with the CEGs, we created a set of non-CEGs by considering genes that did not give rise to any *cis*-eQTL in either cross. To be consistent with the CEG set, only non-CEGs falling in the nIBD-BXH were considered. Thus, a combined set of 2,705 distinct non-CEGs was created by taking the intersection of the genes that did not show *cis*-acting eQTLs in either of the two crosses. It is of note that some of the non-CEGs defined here may show up as CEGs in other segregating mouse populations, in other tissues, or in other F_2 _populations constructed from B6, DBA, and C3H mice (increased number of mice in a F_2 _population would have higher power to detect eQTLs). As additional comprehensive sets of CEGs become available, the sets of CEGs and non-CEGs can be refined to produce more accurate positive as well as negative sets.

### Fraction of CEGs containing cis-SNPs is significantly higher compared to non-CEGs

CEGs by definition are expected to contain genetic variations near their physical location on the genome which give rise to variations of their mRNA levels in a segregating population. We have therefore studied the frequency and density of SNPs in the promoters and non-coding regions (for definitions see below) of the CEGs and compared them to non-CEGs. These studies are described below.

In defining the promoters and non-coding regions, the gene boundaries and exons were first determined based on clustering of all mRNAs and cDNAs (including ESTs) aligning to a common genomic locus as described in detail earlier [22,23] (see Methods). The promoter regions were then defined to be the 5 Kb or 2 Kb sequence upstream of the gene start coordinates. The non-coding regions comprised of the introns and 5 Kb sequence downstream of the genes. SNPs in the promoter and non-coding regions of genes are referred to here as *cis*-SNPs.

Although transcriptional regulatory elements are often found to be concentrated in the immediate promoter region, they are also located in the introns and downstream regions [24]. On one hand examining only the promoter sequence would clearly be insufficient; on the other hand including the introns and down-stream sequences could dilute the density of regulatory elements (if in fact they were enriched in the immediate promoter regions of most genes under consideration in our study), thereby making it difficult to identify any relationship between SNPs and these elements. Therefore we analyzed the promoter and non-coding regions (NCR) separately. In addition to the immediate vicinity of the genes, regulatory elements such as enhancers or silencers can also be present at distances that are far away from the genes themselves [25]; we have not addressed these in our present study.

We analyzed *cis*-SNPs in regions that were most conserved between the mouse and human genomes. Functional non-coding sequences are often assumed to be under evolutionary selection pressure, and thereby conserved relative to the surrounding non-functional sequence. Consequently, phylogenetic footprinting has been widely used for the analyses of non-coding regulatory sequences [26-29]. Although phylogenetic footprinting methods have limitations (sequences from organisms that are too distant or too close can be uninformative), the alignments of rodent-human sequences have been demonstrated in many studies to be successful in identifying regulatory elements, and significant enrichment of known regulatory elements have been found in these regions [26-30]. We therefore investigated the presence of SNPs in the mouse-human aligned regions in the promoters and non-coding regions to see if a higher fraction of CEGs contain *cis*-SNPs in these conserved sequences. For this purpose the mouse-human genome alignments were taken directly from the UCSC genome annotation project [31], where the two genomes were aligned using the BLASTZ software [32] and post-processed to obtain the best alignments for each region (see Methods for details). These alignments represent the most conserved sequences between the mouse and human genomes and cover ~6% of the mouse genome, which is roughly the percentage of mammalian genome that is estimated to be under purifying selection [33].

A significantly higher fraction of the CEGs contained *cis*-SNPs (at p < 0.01 with Fisher exact test, Table 1) compared to non-CEGs. When we considered *cis*-SNPs contained only within regions that are conserved between mouse and human, the fraction of CEGs containing *cis*-SNPs was still observed to be higher than non-CEGs (p < 0.01, Table 1), but the significances were decreased for the conserved promoters regions (p ~10^-3^) compared to all promoter regions (p ~10^-12^). A ratio of over-representation (ROR) for CEGs containing *cis*-SNPs may be defined as the ratio of the fraction of CEGs containing *cis*-SNPs to the fraction of all genes (CEGs+non-CEGs) containing *cis*-SNPs (Table 1, last column). The ROR values were decreased when considering *cis*-SNPs in the conserved promoter regions relative to all promoter regions. Therefore the decreased significance of CEGs containing SNPs in the conserved regions of the promoters could be explained by the decreased ROR value. Another reason contributing to the decreased significance could be the smaller sample size, given many fewer genes contained *cis*-SNPs in conserved regions.

### Higher density of SNPs in promoters and non-coding regions of CEGs

Next, we compared the *cis*-SNP density in the promoters and non-coding regions of CEGs to non-CEGs. Genes with no *cis*-SNPs in their promoters or non-coding regions were ignored for this analysis, since the absolute numbers of genes containing *cis*-SNPs were already compared earlier (Table 1) (consideration of genes with no *cis*-SNPs will only increase the significance of the p-values in Table 1, since a higher fraction of the CEGs contain *cis*-SNPs compared to non-CEGs). *Cis*-SNP densities between the two sets were compared using the non-parametric Wilcoxon rank sum test (Table 2). A non-parametric method was used because the distributions under study were non-normal. A significantly higher density of *cis*-SNPs (number of SNPs per Kb of total non-coding or promoter sequence) was observed in CEGs compared to non-CEGs (p < 0.01).

In order to compare the density of *cis*-SNPs in the conserved and non-conserved regions, genes were partitioned into two sub-sets, namely, those with no *cis*-SNPs in mouse-human conserved regions (subset 1), and those containing *cis*-SNPs in the conserved regions (subset 2) (Table 2). In subset 1, containing a majority of the CEGs, higher *cis*-SNP density was observed in both promoter and non-coding regions (p < 0.01). In subset 2, a higher *cis*-SNP density was observed in non-coding region (p < 0.01) only. Upon normalizing the number of SNPs by the length of the conserved or non-conserved sequence (instead of the total promoter or non-coding sequence length), significantly higher density was observed in both conserved as well as non-conserved non-coding region (p < 0.01, Table 2, subset 2). In the 5 Kb upstream promoter regions of genes in subset 2, significantly higher SNP density was observed only when the number of *cis*-SNPs in mouse-human aligned sequences was normalized by the length of these conserved regions.

### A higher fraction of CEGs has cis-SNPs that alter predicted transcription factor binding sites

In an attempt to study what effect the *cis*-SNPs in CEGs have on the transcription regulatory machinery, the perturbation of transcription factor binding sites (TFBSs) by *cis*-SNPs was investigated. All known mouse, rat and human TFBSs (a total of 2,528 sites) from the TRANSFAC^® ^database [34] were first mapped to the mouse genome using BLASTN (for mapping details see Methods). However, none of the mapped sites overlapped with *cis*-SNPs of any of the CEGs. Consequently we investigated the overlap of *predicted *TFBSs with *cis*-SNPs.

The rationale and caveats for using predicted TFBSs are discussed below. It has been shown through experiments that the score of a transcription factor (TF) binding site, as computed from a position weight matrix (PWM) built from a collection of its known sites, can give a fairly accurate estimate of the *in vitro *DNA binding affinity of the transcription factor to that site (e.g. [35-37]). This observation and the thermodynamic principles behind it forms the basis of most of the generic bioinformatic methods that are in use today to predict TFBSs in genomic DNA (Reviewed in [36,38]). Compared to *in vitro*, the TF-DNA binding events are definitively more complicated *in vivo *since TF binding to DNA in eukaryotes is context dependant (e.g. dependant on other TFs which bind nearby DNA sites, local DNA structure), and influenced by factors like chromatin remodeling and concentration of the TF. But such contextual and other relevant information are available only in rare cases and cannot be generally leveraged in the prediction of TFBSs [38]. Therefore, although the change in TFBS score may not be accurately predictive of the binding of a transcription factor to its target DNA site (and of the change to the target gene's expression) *in vivo*, in the absence of other specific information such as chromosomal regions that are open for the regulatory proteins to bind, the DNA binding partners for a given TF, concentration of TF etc., the approach we have taken here (i.e. looking for base changes that lead to perturbation of the binding sites predicted with models built out of previously known sites for TFs) is a reasonable strategy (and the only *generic *strategy at this time) that one can use to examine how SNPs may affect TF binding to putative TFBSs. This is a common strategy that has also been used by others for the prediction of TFBSs as well as prediction of putative regulatory SNPs that could perturb TF-DNA binding and cause changes to expression of the target gene [39-42].

In our study TFBS predictions were made with PWMs representing the transcription factor DNA binding sites available from the TRANSFAC^® ^(v. 6.3) database [34] using the MATCH™ software [43]. Only PWMs generated from the collections of vertebrate DNA binding sites were used. 30 bp regions were taken around all *cis*-SNPs (a total 61 bp including the SNP nucleotide) and scored with the PWMs (for details see Methods). Both the B6 and DBA alleles were scored, since as explained earlier, these were the variants that were used in the analysis of data from both crosses. If a *cis*-SNP location overlapped with a predicted binding site, and a difference was observed in the predicted binding site score due to the two alleles, the change in score was noted, and the predicted TFBS was considered to be perturbed by the SNP.

Since the TFBSs are typically short and degenerate, predictions using PWMs are known to contain a large percentage of false positives [28,36,44]. Therefore, orthogonal data such as co-regulation of the target genes with the transcription factors or phylogenetic footprinting, are commonly used to increase the specificity of these predictions [28,44,45]. Although the transcription factors and their target genes may not co-regulate at the mRNA level, it is generally assumed that genes that *do *co-regulate across a diverse set of conditions may belong to the same regulatory pathway [46-48]. To reduce the number of false positive predictions for TFBSs we employed a similar strategy, requiring that the transcript levels of the TFs and their putative target genes (based on the TFBS predictions) be significantly correlated across a diverse set of mRNA profiling experiments (see Additional files 3 and 4). Using expression profiles available from a set of 145 diverse mouse tissues and cell-lines [49,50] (referred to here as the 'body-atlas' data set), we determined the Spearman rank-order correlation (with p < 0.01) between all genes and the 282 distinct vertebrate transcription factors in the TRANSFAC^® ^(v. 6.3) database which have known gene symbols as well as PWM models for their DNA binding sites. It is of note that the body-atlas expression data set was used instead of the BXD and BXH F_2 _populations given others have shown that significant correlation between any two genes in a segregating population can result from closely linked eQTLs as opposed to biologically relevant co-regulation [11]. These effects can be amplified in cases where genes give rise to strong *cis*-eQTLs.

**Figure 2:**
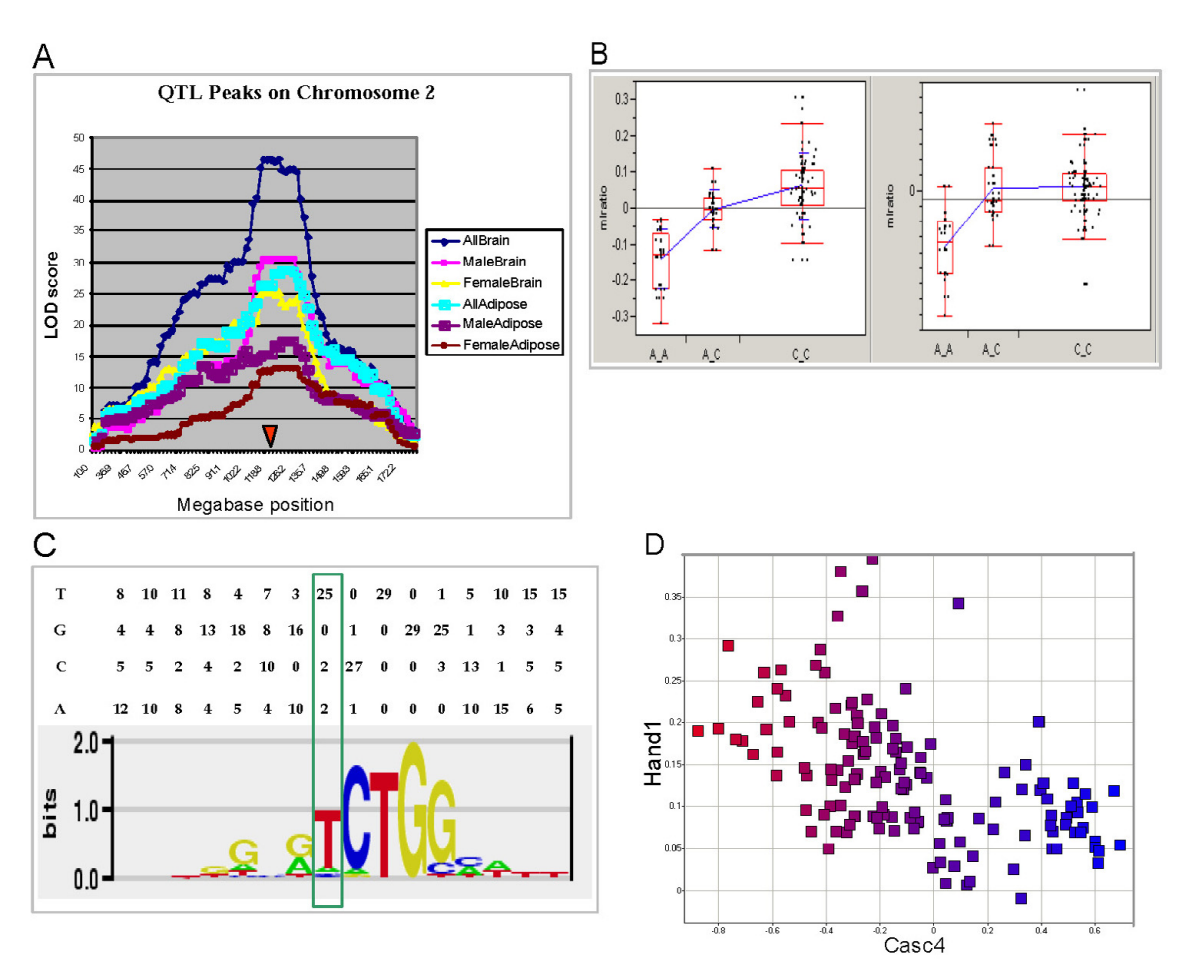
**An example of a putative candidate *cis*-regulatory SNP affecting transcription in mouse F_2 _population**. **A**. *cis*-acting LOD scores for Casc4 on chromosome 2 in multiple tissues and sample sets (male, female or combined/all) in the BXH cross. x-axis – genomic location in Mb, y-axis – LOD score from interval mapping. Physical location of the gene is indicated with a red arrow-head. Only LODs scores >10 are shown. **B**. Association of expression levels of Casc4 with genotypes of the promoter SNP, mCV23866990. The distribution of the expression levels in brain (left) and adipose (right) is shown according to the genotypes of this SNP in the F_2 _animals. A_A represents the DBA and C3H allele, and C_C the B6 allele. **C**. A binding site for transcription factor Hand1 is affected by SNP mCV23866990. The polymorphism changes a highly conserved base in the binding site (T→C change on the reverse strand, boxed and shaded). The frequency matrix and a sequence logo of the profile representing the binding site are shown. **D**. Scatter plot of Casc4 (x-axis) versus Hand1 expression levels in the body atlas data set [49, 50]. Hand1 expression is correlated to that of Casc4 with a p-value of < 10^-6 ^(Spearman rank order correlation -0.58).

We found that a higher fraction of CEGs have *cis*-SNPs affecting predicted TFBS scores in non-coding regions (p = 4.23 × 10^-4^, as determined by the Fisher exact test) (Table 3), when correlations were required between TFs and their target genes (based on TFBS predictions, see Figure 2 for an example). Significance was not observed when the TFBS predictions were *not *filtered by correlations (data not shown), which may be due to the large false positive rate in the predicted TFBS set. Interestingly, the p-value of the hypothesis that more CEGs harbor *cis*-SNPs that disrupt predicted TFBSs (p = 4.23 × 10^-4^) is much larger compared to the p-value of hypothesis that more CEGs contain *cis*-SNPs in the promoter and non-coding region (p ~10^-12^, Table 1). Possible reasons for this observation include: 1) DNA binding sites for most vertebrate TFs cannot be predicted since PWM models for their binding sites are not available, 2) a large fraction of the *cis*-SNPs are neutral with respect to their effects on the transcriptional levels [51], and 3) *cis*-SNPs could perturb regulatory elements other than TFBSs (see Discussions for more details).

In order to determine whether the *cis*-SNPs in CEGs perturb predicted TFBSs with an increased frequency relative to the non-CEGs, we compared the fraction of *cis*-SNPs affecting TFBSs in CEGs versus non-CEGs using the Fisher exact test. The fraction of *cis*-SNPs in CEGs affecting predicted TFBSs was not observed to be higher (at the 0.01 significance level), suggesting that a higher rate of TFBS perturbation by SNPs in CEGs is likely due to the increased density of *cis*-SNPs in these genes relative to non-CEGs.

### An example cis-eQTL and putative regulatory cis-SNP

To illustrate how high-density SNP data may be intersected with eQTL data to identify putative candidate quantitative trait nucleotide (QTN) underlying the eQTLs (and also to illustrate the different types of data we have used in our analyses), we highlight one example of a CEG with a *cis*-SNP in its promoter region perturbing a predicted TFBS (Figure 2). The gene Casc4 (cancer susceptibility candidate 4) gives rise to a strong *cis*-acting eQTLs (LOD score ≥ 10) in a number of tissues in the BXH cross (Figure 2a). As Casc4 is in a nIBD-BXH region, the polymorphisms between B6 and C3H in this region should be identical to those between B6 and DBA. There are five SNPs in the promoter (upstream 5 Kb) of this gene; only one SNP (mCV23866990), which is located close to the 5' end of Casc4 (-701 bp), perturbs the predicted binding-site for a transcription factor, Hand1 [52], whose mRNA level is correlated with that of the gene. The mCV23866990 SNP genotype shows a significant association with the expression level of Casc4 (p < 0.0001 using a standard one-way ANOVA) (Figure 2b). A conserved position in the binding site for the Hand1 is perturbed by mCV23866990 (Figure 2c). By affecting the Hand1 binding site in the promoter, this *cis*-SNP could be responsible for differential expression of Casc4 in the BXH F_2 _population.

Isolating the specific causative regulatory mutations underlying eQTLs that are responsible for variation of gene expression in a segregating mouse population is difficult. This is especially true in an F_2 _population, where regions of linkage disequilibrium are very large in any given region (given an F_2 _population is constructed from intercrossing a single F_1 _founder). Determination of the actual functional role of the causative polymorphisms is even more challenging, since there are several different molecular mechanisms through which mRNA levels in cells can be regulated. Although such challenges exist, *putative *candidate polymorphisms that affect transcription of a given gene may be prioritized for experimental validation, and hypotheses can be generated for the possible biological roles of candidate regulatory *cis*-SNPs based on examination of the data, as illustrated by the example above. For such candidates the gold standard is to introduce the polymorphism in question onto the background of a wild-type mouse and then compare changes in the *in vivo *activity of the gene and phenotypes to the wild-type mouse.

## Discussion

### Cis-SNPs in genes showing cis-acting linkage in segregating mouse populations

SNPs are often used as markers for disease, and as noted earlier, there are now several examples where *cis*-regulatory variants are associated with disease [5,13,14,42,53,54]. Computational approaches for identifying the *cis*-regulatory polymorphisms would therefore be useful in prioritizing the candidate polymorphisms that a play causative role in disease, reducing the laborious experimental process of testing multiple candidate variants *in vivo*, selecting biologically meaningful SNPs for association studies, and ultimately in generating testable hypotheses for elucidating the molecular basis of a given disease. However, little bioinformatics research has been done in a systematic way to build predictors of variations that are likely to affect gene-expression in segregating mammalian populations [39-42,55]. In this study we have investigated the frequency and potential biological role of the polymorphisms underlying genes whose expression give rise to strong *cis*-linkage in segregating mouse populations. The study provides the first investigation of putative regulatory SNPs around genes showing *cis*-linkage, and insight into the challenges associated with identifying the causative regulatory variants in such populations through bioinformatic sequence analyses methods. In addition, the data we provide here (CEGs from four different tissues in two crosses, *cis*-SNPs, and predicted TFBSs affected by those *cis*-SNPs) should benefit further investigations in this area.

There have been a few previous studies surveying the role of *cis*-polymorphisms and haplotypes in promoter regions of sets of human genes, and identifying those that change expression [5,40,51,53,56-58]. These studies considered a relatively small sampling of genes (<300) and assessed the promoter SNPs that affected the expression in a limited number of cell lines with reporter gene assays [51,57-59]. Since the changes in expression due to the polymorphisms were tested in cell-lines, it is not known if the SNPs that caused expression changes in the *in vitro *assays are responsible for varying levels of expression *in vivo *in genetically segregating populations. We started with large-scale genetic linkage data of gene expression and investigated the frequency of polymorphisms in the genes showing *cis*-linkage. Therefore the work we present here (namely, investigation of SNPs in the vicinity of genes with *cis*-expression linkage in segregating populations in as many as four different tissues), is complementary to the previous work, and provides a different approach to investigating *cis*-regulatory SNPs with murine populations.

One recent study reports the mapping of *cis*-regulatory variants in a small set of genes to haplotype blocks in human samples [60], and another recent study reports the investigation of *cis*-regulatory variations in 3' UTRs of a set of genes showing *cis*-acting regulation in a panel of mouse recombinant congenic strains [61]. However, to the best of our knowledge, the present study represents the first large scale genome-wide survey of *cis*-SNPs in genes that give rise to strong *cis*-eQTL in a mammalian population, and an investigation of their potential role in disrupting putative *cis*-regulatory elements. We observe a significantly higher fraction of CEGs to contain *cis*-SNPs compared to non-CEGs, and that the density of these SNPs is significantly higher in the CEGs. We have not conclusively proven the functional role of any polymorphism in regulating the expression of CEGs through *in vitro *or *in vivo *experimental validation, and many of the SNPs in the vicinity of the CEGs could be neutral (i.e. have no consequence on the expression levels). However, CEGs by definition should have variations near the genes themselves affecting their expression in a segregating population, and based on earlier work on human genes with promoter polymorphisms, it has been estimated that a sizable fraction of *cis*-variants (about one third of the SNPs in the promoters) may alter gene-expression [5,51]. Therefore, it is reasonable to infer that the higher density of *cis*-SNPs in CEGs is associated with changes in expression of those genes, and one or more of the *cis*-SNPs would be responsible for causing variation of expression in a large fraction of the CEGs in the mouse F_2 _populations, although we do not exactly know how many causal regulatory *cis*-SNPs are in this set of CEGs.

We investigated the effect of a few other relevant biological factors which could give rise to *cis*-eQTLs in our data-set instead of (or in addition to) *cis*-SNPs in the promoters and non-coding regions, such as non-sense mediated decay (NMD), polymorphisms in exons, and genomic segmental duplications. We estimated that a very small fraction of our set of CEGs may arise due to NMD and segmental duplications (see  Methods section for details). A higher fraction of CEGs was seen to contain SNPs in their exons (1081 out of 2047, p = 2.5 × 10^-12 ^with Fisher exact test) relative to the non-CEGs. But an increased fraction of the CEGs that contained exonic SNPs also contained promoter and non-coding *cis*-SNPs relative to non-CEGs (p < 10^-4 ^with Fisher exact test). Therefore, we did not exclude these genes from the analyses presented here (the purpose of which was to investigate potential regulatory SNPs in the non-coding and promoter regions). It is worth noting however that our analyses with the set of CEGs and non-CEGs which did not contain any exonic SNPs yielded results that were very similar to those obtained from the full set of CEGs and non-CEGs.

One of our objectives was to investigate the challenges involved in identification of the causative *cis*-regulatory SNPs through bioinformatic sequence analysis approaches. To that end, we have examined the propensities of the SNPs in potentially functional non-coding sequences (namely, mouse-human conserved sequences) and predicted transcription factor biding sites. A few recent studies have suggested the use of mouse-human alignments to identify putative candidate regulatory SNPs [40,41], and although SNPs falling in these conserved regions have been shown to affect transcription [40], it is unclear whether a majority of the regulatory variants lie in these regions. In our analyses we find that the sequence variations around the CEGs are not specifically enriched in evolutionarily conserved non-coding and promoter sequences, and in fact in the majority of the CEGs all of the SNPs are outside of these highly conserved regions. It is possible that the causative regulatory SNPs lie further away in these CEGs (sequences that are > ± 5 Kb away from the genes) where they alter conserved regulatory elements (such as silencers or enhancers). However, the higher *cis*-SNP density in the immediate vicinity of these CEGs (relative to non-CEGs) suggests that a significant fraction of *cis*-regulatory SNP could lie outside of regions that are most conserved in mammalian evolution. As comparative genomics and phylogenetic footprinting approaches are frequently utilized in the searches for functional regulatory elements in mammalian genomes, and computational prediction of transcriptional regulatory elements is a difficult problem [28,36,44], the above observations imply that the identification of *cis*-regulatory variations in genetically segregating populations is likely to be difficult using sequence-driven bioinformatic approaches alone. Since the information for transcriptional regulatory networks is in part hard-wired in the genomic DNA itself through the array of regulatory elements [62], one can hypothesize that for CEGs where the *cis*-regulatory SNPs are not present in the highly conserved promoter or non-coding sequences, variations of expression may not cause a significantly perturbation of the transcriptional networks that have been conserved in the mammals. Experimental validation of a set of SNPs affecting putative regulatory sites in conserved and non-conserved regions (both in the immediate vicinity of the genes, and also some distances away), will be ultimately required to fully understand how the in *cis*-SNPs affect gene expression in segregating populations. This would consequently lead to a better understanding of the bioinformatic approaches that would be effective in identifying *cis*-regulatory variants.

Below we describe the examination of a set of characterized human *cis*-regulatory SNPs that are associated with inherited diseases. A collection of these rare examples is available from the rSNP_Guide database [42,63]. From the examples presented in this database (see [63]) we collected a set of 33 *cis*-regulatory SNPs in 5 distinct genes (PROC, TNF, HGB2, GP1BB, and F7) that are: a) known to be underlying or associated with inherited human diseases, b) known or predicted to disrupt transcription factor binding sites, and c) have flanking sequences available from the Human Gene Mutation Database [64] so that they could be used for mapping reliably to the human genome assembly. An examination of the locations of these regulatory SNPs indicates that 10 SNPs in 2 genes are in the human-mouse conserved regions, while the remaining 23 SNPs in 3 genes are in non-conserved regions. In addition to the above cases that were taken directly from rSNP_Guide database, from two recent publications we examined the non-coding SNPs (three in total) within or around two genes, namely INSIG2 [65] and TCF7L2 [66], that represent extremely rare examples of variants associated with complex disease (obesity and diabetes respectively in this case) that have been validated across diverse and multiple human cohorts. These causative SNPs were also outside of human-mouse conserved regions. This is a small sample of genes to draw concrete conclusions from; however this observation with the well characterized human SNPs supports our similar finding from the mouse data and suggests that a large fraction of the causative *cis*-regulatory SNPs, including those that are associated with inherited disease, could be outside of the sequences that are highly conserved in mammalian evolution.

### Perturbation of putative transcription factor binding sites by cis-SNPs

A higher fraction of CEGs had TFBS predictions perturbed by SNPs in the non-coding region. No significant difference was observed in the fraction of total SNPs affecting binding sites between CEGs and non-CEGs, suggesting that the higher fraction of perturbations of TFBSs in the CEGs was a consequence of increased *cis*-SNP density.

Several factors are likely to have confounded our study with predicted TF binding sites: 1) it is possible that the false positive rate of TFBS prediction is high, even after requiring correlations of TFs to putative target genes; 2) at the time of our analysis binding site models for only ~300 vertebrate TFs were available from the TRANSFAC database, whereas the number of distinct TFs in mammals are estimated to be around 2,000 [33]; therefore prediction of DNA binding sites for the majority of TFs was not possible; 3) although often enriched in the immediate promoter region, transcription regulatory elements in mammals can be spread over large distances (sometimes more than 100 Kb [25]) whereas in this present study we considered only the immediate vicinity of the genes (± 5 Kb); 4) transcriptional regulatory elements frequently act in conjunction with others forming regulatory modules where multiple TFs bind DNA (involving both protein-DNA as well as protein-protein interactions); consequently the change in score of one individual binding site by an overlapping *cis*-SNP may often fail to reflect the extent to which transcription is affected by that mutation. It would clearly be useful to re-analyze the data when significantly more experimentally verified murine transcription factor binding sites are available in order to obtain a better understanding of how *cis*-variations specifically affect the transcriptional machinery, and whether a larger fraction of SNPs in the CEGs perturb known binding sites.

It is of note that in a recent study of *Saccharomyces cerevisiae *segregants it was shown that genes having *cis*-linkage contained a higher frequency of SNPs in promoters and 3' UTR sequences [12]. The study also found moderate evidence for enrichment of SNPs in TFBS sequences that were mapped using the ChIP-chip technology [67]. The yeast study with TF binding sites was not confounded by some of the factors mentioned above, since most of the regulatory sequences were experimentally determined, and a comprehensive set of DNA binding sequences for almost all of the yeast TFs were available [67].

Obviously, in addition to TFBSs, other classes of regulatory sequences, e.g. those affecting transport from the nucleus, mRNA stability or decay, RNA mediated regulation, and those potentially involved in epigenetic regulation of gene expression, could be affected by *cis*-SNPs, which we have not studied here. Coding SNPs that cause changes in the protein structure can act in *trans *to influence expression through a feedback loop as shown recently for the AMN1 gene in *S. cerevisiae *[12]. Such cases were also not studied here.

### Undetermined factors in our study

The specificity of the identification of *cis*-acting eQTLs was unknown. Recently Doss et al. [11], gives a lower bound estimate of the true positive rate for the BXD cross (64%); we have used more stringent thresholds for identification of putative *cis*-eQTLs in our study to increase the true positive rate, so we anticipate the true positive rate would be higher than 64%, but the exact number is not known. The specificity of TFBS predictions is unknown; moreover binding sites for the majority of the TFs could not be predicted because TRANSFAC^® ^PWMs were unavailable. It is likely that additional SNPs exist between the strains we studied that had not been identified in the databases we used in our study [68]. Even with these unknown factors in our current study, several observations have been made that shed light on the nature of variation in genes showing *cis*-linkage in segregating populations, as well as the bioinformatic challenges that are involved in characterizing the non-coding *cis*-regulatory polymorphisms using computational sequence analysis strategies.

### Future work

The problem of identifying and annotating the functional *cis*-regulatory polymorphisms is a difficult one that will require various experimental as well as computational approaches to address. Our understanding of *cis*-regulatory variations and their biological role would benefit from *in-vivo *experimental evaluation of the contribution of polymorphisms around CEGs towards changes in gene expression, characterization of more regulatory elements in the genome (which is severely limited at this time), examination of the multi-species genome alignments, and more accurate prediction of the (transcriptional and other) regulatory elements. The data on CEGs and *cis*-SNPs that we supply here (supplementary information) will provide a valuable resource for further exploration in this area.

## Conclusion

The analyses of *cis*-SNPs in the promoters and non-coding regions around *cis*-acting eQTL genes (CEGs) in mouse F_2 _populations indicate that a significantly higher fraction of CEGs contain *cis*-SNPs compared to non-CEGs. CEGs also contain higher SNP density in the promoters and non-coding sequences relative to the non-CEGs. Since non-coding sequences that are conserved in mammalian evolution are often biologically functional, the propensity of *cis*-SNPs in the promoter and non-coding regions that are most conserved between mouse and human was examined. A majority of the CEGs having *cis*-SNPs did not contain any *cis*-SNP in these conserved regions, and in the CEGs that contained *cis*-SNPs in conserved regions, the enrichment of *cis*-SNPs occurred both in conserved as well as non-conserved regions. This suggests many of the *cis*-regulatory SNPs underlying eQTLs and responsible for causing gene-expression changes in segregating populations could lie outside of the sequences that are most highly conserved in mammalian evolution. To investigate the possible biological role of the *cis*-SNPs in disrupting the transcriptional regulatory elements, we studied the perturbation of the predicted transcription factor binding sites (TFBSs) by the *cis*-SNPs. Relative to non-CEGs, a significantly higher fraction of CEGs harbor *cis*-SNPs that perturb the predicted TFBSs. However the fraction of *cis*-SNPs in the CEGs affecting the binding sites is not higher, suggesting that the increased incidence of TFBS perturbation in the CEGs is due to the higher *cis*-SNP density. These observations imply that the identification and annotation of *cis*-regulatory variations in genetically segregating populations is likely to be difficult using sequence-driven bioinformatic approaches alone.

## Methods

### Genomic data: Mouse genome assembly, gene sets, SNP locations, genomic regions that are IBD between strains, and mouse-human conserved regions

The UCSC mouse genome assembly mm4 [31] (NCBI build 32) was downloaded and used for all mapping purposes. All mouse mRNAs, cDNAs and ESTs were aligned to the mm4 assembly and clustered to produce gene and exon coordinates as described in detail previously [22,23] (for gene and exon coordinates see Additional file 5). Celera (release 3.4) [20] and public reference (dbSNP build 120 [21]) mouse SNPs were mapped onto the mm4 assembly using BLASTN as described in [19]. Those SNPs between the strains C57BL/6J and DBA/2J that mapped uniquely to the autosomes, had allele count > 1, and allele frequency ≥ 10%, were used for analysis. SNPs in repeat regions were removed (repeat coordinates in mm4 were downloaded from UCSC annotation server), which left a total of 484,727 Celera and 24,332 dbSNPs. 16,809 SNPs between the Celera and dbSNP databases were identical in genomic location, leaving a unique, non-redundant set of 492,250 SNPs. This data set was used for analyses of *cis*-SNPs.

Genomic regions that are identical by descent (IBD) between mouse strains were taken directly from Cervino et al. [19]. Cervino et al. used a window of 50 Kb which was moved through the genome at 10 Kb intervals; regions in which fewer than five consecutive SNPs were observed between two strains were identified as blocks that were IBD. Genes were taken with 5 Kb flanking regions (± 5 Kb), and if the gene or its flanking region overlapped with regions of IBD, the gene was considered to be in an IBD region.

Mouse (UCSC assembly mm4) and human (UCSC assembly hg16) genome alignments (axtTight track) were downloaded from the UCSC mouse-human alignment download site [69]. In the axtTight track, mouse and human genomes were aligned using BLASTZ [32] and post-processed to obtained the best alignments for each region. The amount of mouse genome covered in axtTight track is ~6%.

### BXD and BXH crosses and mRNA profiling

Both the crosses under study here have been described earlier [15,16]; we simply describe the key features of those crosses in brief here for the benefit of the readers. In the BXD cross, an F_2 _population consisting of 111 mice was constructed from a cross of two inbred strains of mice, C57BL/6J and DBA/2J [10,15]. Only female mice were maintained in this population. At 16 months of age the mice were euthanized and their livers extracted for gene expression profiling. The mice were genotyped at 139 microsatellite markers uniformly distributed over the mouse genome to allow for the genetic mapping of the gene expression and disease traits. The BXH F_2 _mouse population was constructed from C57BL/6J ApoE null (B6.ApoE-/-) and C3H/HeJ ApoE null (C3H.ApoE-/-) mice [16,19]. F_1 _mice were generated from reciprocal intercrossing between B6.ApoE-/- and C3H.ApoE-/-, and F_2 _mice were subsequently bred by intercrossing F_1 _mice. A total of 334 (169 female, 165 male) were bred. Mice were sacrificed at 24 weeks and four tissues (liver, white adipose, whole brain, muscle) were extracted for mRNA profiling. Genomic DNA was isolated from kidney. A linkage map for all 19 autosomes was constructed using 1032 SNPs markers, giving rise to a genetic map with an average density of 1.5 cM. Genotyping was conducted by ParAllele using the molecular-inversion probe multiplex technique [70].

All of the expression data from the two crosses we have used here for eQTL analyses were generated and described previously [6,10,16]. For the BXD and BXH crosses expression measurements were available for 21,740 and 21,640 transcripts, respectively, representing 18,774 and 19,197 distinct coding genes which mapped uniquely to the 19 autosomal chromosomes. 12,597 genes were common between the microarrays used to profile each cross.

Although the actual mRNA profiling experiments were described earlier [10,16], a summary of the method is given below for the reader's information. Total RNA from the BXD and BXH samples was purified from 25-mg portions using an RNeasy Mini Kit according to the manufacturer's instructions (Qiagen, Valencia, CA, USA), as previously described for the BXD set [10]. Fluorescently labeled cRNA (5 mg) from each F_2 _animal in each cross was hybridized against a pool of RNAs specific to each cross. The RNA pools for each cross were constructed from equal aliquots of RNA from all animals in the BXD cross and 150 randomly selected animals in the BXH cross. Array images were processed as previously described to obtain background noise, single-channel intensity, and associated measurement error estimates [71]. Expression changes between two samples were quantified as log_10_(expression ratio), where the "expression ratio" was taken to be the ratio between normalized, background-corrected intensity values for the two channels (red and green) for each spot on the array. An error model for the log ratio was applied as previously described to quantify the significance of differential expression between two samples [71].

### eQTL mapping and identification of CEGs and non-CEGs

Expression level for each gene was treated as a continuous variable and mapped to the genome using interval mapping. QTL mapping in the BXD cross was done as described [6,10]. For BXH, QTL mapping was done with the QTL-Cartographer suite of programs [72] using the established interval mapping [17] procedure. Since each experiment was hybridized in flour reverse pairs, log_10 _of the two expression ratios was taken and averaged to get the expression level (called ml-ratio for mean log ratio) for a gene. When males and females were treated as one combined group (in order to increase the power to detect linkages with increased number of animals), the gender effect on expression was accounted for by subtracting the gender-specific mean from each expression value. Specific thresholds for selecting CEGs are given in the results section.

For some genes, the probes on the microarray, when mapped to the mouse genome (UCSC mm4 [31] or NCBI build 32), overlapped with SNPs. For these genes (which consisted roughly 4.6% of the total number of genes represented on the microarrays), *cis*-eQTLs could simply arise due to polymorphisms in the probe sequences influencing hybridization of the mRNA to the microarray, rather than non-coding *cis*-variations influencing their expression. Such genes were therefore removed from the list, in order to minimize the false positive calls on CEGs.

Nonsense mediated decay (NMD) is a mechanism of mRNA surveillance that ensures rapid degradation of transcripts with premature stop codons [73]. Therefore some CEGs may not have non-coding *cis*-regulatory variation but instead contain nonsense mutations that result in NMD, which is detected as a *cis*-eQTL event. The Celera mouse SNP database [20] (from which most of the SNPs for our analysis of *cis*-variants were taken) provided the annotation on SNPs that cause nonsense mutations; 63 distinct genes represented on the microarrays used for the crosses were annotated as having nonsense mutations. Only 4 CEGs from the BXH cross (~0.1% of all CEGs from that cross) and none of the CEGs from the BXD cross were annotated as having SNPs resulting in nonsense mutations. The enrichment of genes having SNPs annotated as causing NMD in our list of 2,047 CEGs (Table 1) was not significant (p = 0.33 with the Fisher exact test). Since this fraction was small and the p-value not significant, we are confident that NMD did not introduce any bias in our results. In addition, the genes containing nonsense SNPs could still have non-coding *cis*-variations affecting their expression. In our analysis we therefore did not exclude the genes with nonsense SNPs.

In addition to the factors discussed above, variations in segmental duplications in the genome may affect expression. A rough map of genomic duplications is available for the B6 strain [74], however no map of the *variations *of genomic duplications between mouse strains used to construct the F_2 _crosses is available, and it is not known whether significant variations exist between mouse inbred strains in terms of genomic duplications. The analysis that one can do with SNPs (which gives variations between strains) is therefore not possible with the genomic duplications. Nevertheless, we looked to see if CEGs contained an increased number of genes that were in the duplicated regions using the available map of segmental duplications from B6 [74]. From the genomic coordinates of the segmental duplications [74, 75], we obtained the list of genes that were contained within these regions. A total of 123 distinct genes that were represented on our microarrays were within the duplicated regions. We then checked whether these genes are over-represented in the CEGs using the Fisher exact test. Only 11 of the 2,047 CEGs were in regions that underwent segmental duplications, and we found no evidence of enrichment of these genes in our list of CEGs (p = 0.45 with Fisher exact test). Since the maps of duplications in other mouse strains are not available, it is not possible to check whether the CEGs are enriched for genes contained in the duplicated regions of C3H or DBA. It is of note however, even if the duplicated regions in the C3H and DBA did not overlap those in the B6 strain (but the extent of duplications remained roughly the same between the different mouse strains), we would still observe a very small fraction of CEGs to be in these regions.

In addition to checking for the enrichment of genes known to be located within segmental duplication regions in our list of CEGs, we employed a different strategy to check whether a significant fraction of CEGs could arise due to variations in duplications. This analysis was based on the hypothesis that if a certain region on the genome, containing multiple CEGs, was duplicated in one of the parental strains involved in a cross but not the other (i.e. variations of duplication between parental strains), and if this duplication was responsible for giving rise to *cis*-eQTLs in an F_2 _population, then the CEGs contained in the duplicated region would all show the same sign of the additive component of their eQTLs. This is because the F_2 _mice containing the duplicated region would always be expected to have higher levels of mRNA (having multiple copies) for all the genes contained within that region. In other regions of the genome, where duplication was not responsible for differential mRNA levels between the parental strains or the F_2 _animals, the signs of the eQTLs for tandem genes on the genome would be expected to be random, and follow a simple Binomial distribution. In the BxH cross, which contained the majority of CEGs, we observed the instances of 3 or 4 tandem CEGs having the same sign of their *cis*-eQTLs. If the signs of the eQTLs for CEGs were completely random, the probability of 3 tandem CEGs having the same sign of their eQTLs would be 0.25, the probability of 4 tandem CEGs having the same sign of their eQTLs would be 0.125. Using a Binomial distribution we did not observe any deviation from these probabilities in our data (p > 0.1). This suggests that in our set of CEGs, variations in long duplication regions between strains (containing 3 or more CEGs), was not an important factor in giving rise to a significant fraction of the *cis*-eQTLs.

### Description of the IBD map used for analysis

The IBD map used in this study [19] was constructed by looking at 50 Kb sequence windows in the different strains (moving the window through the genome at 10 Kb steps), and identifying as IBD regions the windows that had fewer than five consecutive SNPs [19]. Although this provides a comprehensive IBD map for multiple mouse strains for which complete genome sequences are not yet available, the IBD segments defined in this way are coarse as they have been derived using a ~10,300 SNP genotype map, whereas there are more than 2.5 million SNPs reported in the different mouse strains [20]. While extensive genome sequence coverage is available for the B6 and DBA strains [20,33], allowing for a high-resolution IBD map to be constructed between these two strains, the BXD cross provides a small fraction (338 out of 4,107) of the total CEGs considered in this study. On the other hand, the BXH cross provides a far richer set of CEGs (3,769 out of the 4,107 considered in this study), but the complete C3H (C3H/HeJ) genomic sequence is not available. Therefore, we chose to leverage one of the previously published comprehensive, lower resolution maps based on a consistent set of SNPs genotyped in the B6, DBA, and C3H strains [19, 76]. The utility of the IBD map used here [19] has been demonstrated by its successful application in the identification of a causal disease gene [19], and we anticipate that our conclusions will remain the same (although some of the specific numbers presented here may change) with a finer IBD map that will become available at a later date.

With the IBD map used here [19] (built with a set of around ~10,300 SNPs), we examined the amount of genomic sequence, and the numbers of SNPs and genes falling within and outside of the IBD blocks. Of the 2.5 Gb mouse genomic sequence [33], 1.14 Gb (~45.6%) fell within IBD blocks between B6 and DBA, whereas 1.01 Gb (~40.4%) fell within IBD blocks between B6 and C3H. Of the 492,250 SNPs compiled largely from the Celera mouse sequence database [20] that were outside repeat regions and polymorphic between B6 and DBA, 404,095 (~82.1%) fell in regions that were not in IBD (nIBD). Of the 18,774 autosomal genes represented on the BXD microarray, 10,259 (54.6%) were contained within regions that were nIBD between the two parental strains B6 and DBA (which is according to expectation, since 100–45.6 or 54.4% of the total genomic sequence is in nIBD regions between these two strains, as noted above), whereas 279 of the 338 CEGs (82.5%) were in nIBD (p = 8.87 × 10^-28 ^as determined by the Fisher exact test). Of the 19,197 autosomal genes represented on the microarray for the BXH study, 11,628 (60.6%) genes were in nIBD regions between B6 and C3H (again according to expectation since 100–40.4 or ~59.6% of mouse genomic sequence is in nIBD regions between these two strains), whereas 3,219 out of the 3,769 CEGs (85.4%) were nIBD between these two strains (p = 4.08 × 10^-296^, by the Fisher exact test). The significant enrichment of CEGs in nIBD regions is expected since there should be *cis*-variants near the CEGs that result in *cis*-linkage by altering one or more regulatory elements, and by definition these variants are likely to be largely biased towards the nIBD regions.

### SNPs perturbing transcription factor binding sites (TFBS)

For finding the overlap of known transcription factor binding sites with SNPs, 30 bp sequences around the SNPs (total 61 bp) were taken, and the experimentally determined human, rat and mouse binding sites from the TRANSFAC^® ^database [34] were mapped to these sequences (both B6 and DBA alleles) using BLASTN. Only sites which mapped with a threshold of 95% identity were kept. 16 distinct binding sites mapped in this way overlapped with SNPs between B6 and DBA. However of these, none overlapped with *cis*-SNPs that were in the promoter or non-coding region of any of the genes.

Consequently, transcription factor binding site predictions were made on the 61 bp sequences around the SNPs as described below. Although TFBSs are often short, we took a length of 30 bp on either side of the SNPs, since in the TRANSFAC^® ^database the longest vertebrate position weight matrix was 30 bp. Site predictions were made with the vertebrate position weight matrix models (PWMs) available from the TRANSFAC^® ^database (version 6.3) [34] using the MATCH™ software [43]. The individual TFBS prediction cutoff scores were given by TRANSFAC^® ^(based on an algorithm that minimizes the sum of the false positive and false negative rates of predictions using known sites). With the application of the TRANSFAC thresholds, the scores of individual sites ranged from 0.751 to 1. For each SNP, two sequences were generated containing the B6 and DBA alleles. Both sequences were scored and the change in score of the binding site prediction due to a SNP was recorded. In the analyses we have reported, we did not chose a threshold for the score difference due to a SNP intersecting with a TFBS prediction. Once a binding site was predicted with the threshold given by TRANSFAC, any change to the score of that site was considered as a potential perturbation that could represent an alteration of binding of the TF to that site leading to a change in expression. We also used an increased threshold of 0.01 for score changes to a site by an SNP, and that provided very similar results and identical conclusions (data not shown).

### Correlations between transcription factors and genes from mouse body atlas

Expression profiles of all known mouse genes were determined over 145 tissues and cell lines (called 'Body atlas' data set) as described previously [49,50]. Spearman (rank order) correlations were determined between the mRNA levels of each of the known transcription factors and all other genes and correlates with p-value < 0.01 were stored for analysis.

## Authors' contributions

DG: Conceived the study, performed analyses and wrote the paper

TX: Performed analyses and helped in writing the paper

MA: Performed analyses

SWE: Performed some analyses and provided advice

GL: Provided technical support

SSW: Performed experiments and generated data

EES: Conceived the study, performed analyses, helped in writing of the paper, and secured funding

All authors have read and approved the final manuscript.

## Supplementary Material

Additional file 1 All genes showing *cis*-expression linkage (CEGs) in the two mouse crosses. The mouse cross, the tissue in which the *cis*-linkage was observed, LocusLink identifier, official gene symbol, physical locations of the genes (UCSC mm4 or NCBI build 32 assembly), and the *cis*-acting LOD scores are given. Click here for file

Additional file 2 The complete integrated list of CEGs from BxD and BxH crosses. Gene coordinates are with respect to UCSC mm4 assembly (NCBI build 32). The *cis*-acting eQTL information of these genes may be obtained from the Additional file 1. Click here for file

Additional file 3 Comprehensive set of promoter *cis*-SNPs in CEGs. All *cis*-SNPs in the promoter (upto 5 Kb upstream gene) of the CEGs, along with their location in conserved mouse-human regions (indicated by 'M-H CONS' in the CONSERVATION column), the predicted transcription factor binding sites perturbed by the SNP, and correlation of the gene to the transcription factors (if p < 0.01) (in cases where there is a transcription factor binding site present that is perturbed by a *cis*-SNP) are given. All Celera SNPs [20] are now also available through latest release of the public mouse dbSNP database [21]. The public dbSNP identifiers corresponding to the Celera SNPs are therefore provided for reference. Click here for file

Additional file 4 Comprehensive set of *cis*-SNPs in non-coding regions of CEGs. All SNPs in the non-coding region (introns and 5 Kb downstream) of the CEGs, along with their location in conserved mouse-human regions (indicated by 'M-H CONS' in the CONSERVATION column), the predicted transcription factor binding sites perturbed by the SNP, and correlation of the gene to the transcription factors (if p < 0.01) (in cases where there is a transcription factor binding site present that is perturbed by a *cis*-SNP) are given. dbSNP identifiers corresponding to the Celera SNPs are provided. Click here for file

Additional file 5 Gene structure of the CEGs. Exons of all 2,047 CEGs mapped to the UCSC mm4 assembly (NCBI build 32) are given. This data helps in finding where a non-coding *cis*-SNP is located in the gene. Click here for file

## Figures and Tables

**Table 1:** Genes containing SNPs in promoters (Prom) and non-coding regions (NCR)

**Region**	**Number of Total Genes (CEGs+Non-CEGs)**	**Number of CEGs**	**Total Genes Containing SNPs**	**CEGs Containing SNPs**	**P-value (FET)**	**Ratio of Over-representation**
NCR	4752	2047	3514	1769	**6.03E-12**	1.169
Prom 2 Kb			1569	863	**2.48E-12**	1.277
Prom 5 Kb			2260	1220	**3.50E-12**	1.253
Cons NCR			1476	782	**4.20E-12**	1.230
Cons Prom 2 Kb			236	122	**2.60E-03**	1.200
Cons Prom 5 Kb			388	196	**8.79E-04**	1.173

CEG and non-CEG sets are defined in the text. Data for genes containing SNPs in non-coding region (NCR), 2 Kb upstream (Prom 2 Kb) and 5 Kb (Prom 5 Kb) upstream regions are given. Data on genes containing SNPs in conserved regions between mouse and human are indicated by 'Cons'. p-values less than 0.01 are in bold. All p-values are based on the Fisher exact test (FET). The ratio of over-representation (ROR) is defined as: ratio of the fraction of CEGs containing *cis*-SNPs to the fraction of all genes (CEGs+non-CEGs) containing *cis*-SNPs.

**Table 2:** SNP density in the promoter or non-coding regions of CEGs and non-CEGs

**Gene Set**	**Region**	**Number of CEGs**	**Number of Non-CEGs**	**SNP Density** **(Normalized by total non-coding or promoter length)**			**SNP Density** **(Normalized by Conserved or Non-Cons region length)**		
					
				**Mean SNP Density CEGs**	**Mean SNP Density Non-CEGs**	**WRST** **p-value**	**Mean SNP Density CEGs**	**Mean SNP Density Non-CEGs**	**WRST** **p-value**
Full Combined Set	NCR	1769	1745	0.630	0.463	**< E-12**	NA	NA	NA
	Prom 2 Kb	863	706	1.291	1.172	**0.008**	NA	NA	NA
	Prom 5 Kb	1220	1040	0.789	0.685	**2.70E-06**	NA	NA	NA

Genes with No	NCR	987	1051	0.546	0.352	**< E-12**	NA	NA	NA
SNPs in Conserved		Prom 2 Kb	741	592	1.243	1.106	**0.009**	NA	NA	NA
Regions (subset 1)	Prom 5 Kb	1024	848	0.731	0.620	**1.50E-06**	NA	NA	NA

Genes having SNPs	All NCR			0.736	0.630	**5.00E-10**	NA	NA	NA
in Conserved	Non Cons NCR	782	694	0.657	0.554	**9.00E-09**	0.732	0.620	**1.00E-08**
Regions (subset 2)	Cons NCR			0.091	0.101	0.050	1.359	1.193	**2.71E-06**
	All Prom 2 Kb			1.581	1.513	0.172	NA	NA	NA
	Non Cons 2 Kb	122	114	1.376	1.415	0.498	1.886	2.169	0.482
	Cos Prom 2 Kb			0.758	0.706	0.278	5.227	5.721	0.026
	All Prom 5 Kb			1.091	0.967	0.036	NA	NA	NA
	Non Cons 5 Kb	196	192	0.910	0.822	0.119	1.139	1.025	0.174
	Cons Prom 5 Kb			0.357	0.317	0.036	3.763	3.276	**0.001**

"SNP density (Normalized by total non-coding or promoter length)" = 1000*(total number of SNPs in non-coding or promoter sequence)/(total non-coding or promoter length). "SNP density (Normalized by Conserved or Non-Cons region length)" = 1000*(the number of SNPs in conserved or non-conserved regions)/(total length of the conserved or non-conserved sequence in promoters or non-coding regions). "Mean SNP density" gives the average SNP-density over all the genes in a particular set. The means are only given for reference, and have not been used for calculation of p-values (which were done using a non-parametric method). p-values of significance with the Wilcoxon rank sum test (WRST) are given. H_0 _= CEGs and non-CEGs have equal SNP density, H_A _= CEGs have higher SNP density compared to non-CEGs. p-values less than 0.01 are in bold.

**Table 3:** Perturbation of TFBSs by *cis*-SNPs and summary gene counts where TFBS predictions are affected by *cis*-SNPs

**Region**	**Total Genes Containing SNPs**	**CEGs Containing SNPs**	**Total Genes with TFBS Score Changed by SNPs**	**CEGs with TFBS Score Changed by SNPs**	**P-value (FET)**
NCR	3514	1769	610	344	**4.23E-04**
Prom 2 Kb	1569	863	94	61	0.0174
Prom 5 Kb	2260	1220	193	111	0.1346
Cons NCR	1476	782	129	66	0.6338
Cons Prom 2 Kb	236	122	12	8	0.0852
Cons Prom 5 Kb	388	196	27	15	0.2293

The total gene-set consisted of the combined set of CEGs and non-CEGs as described in the text. All p-values are based on the Fisher exact test (FET); p-values less than 0.01 are in bold.

## References

[B1] Chakravarti A (2001). To a future of genetic medicine. Nature.

[B2] Knight JC (2005). Regulatory polymorphisms underlying complex disease traits. J Mol Med.

[B3] Wang X, Tomso DJ, Liu X, Bell DA (2005). Single nucleotide polymorphism in transcriptional regulatory regions and expression of environmentally responsive genes. Toxicol Appl Pharmacol.

[B4] Pastinen T, Hudson TJ (2004). Cis-acting regulatory variation in the human genome. Science.

[B5] Buckland PR (2004). Allele-specific gene expression differences in humans. Hum Mol Genet.

[B6] Schadt EE, Lamb J, Yang X, Zhu J, Edwards S, Guhathakurta D, Sieberts SK, Monks S, Reitman M, Zhang C, Lum PY, Leonardson A, Thieringer R, Metzger JM, Yang L, Castle J, Zhu H, Kash SF, Drake TA, Sachs A, Lusis AJ (2005). An integrative genomics approach to infer causal associations between gene expression and disease. Nat Genet.

[B7] Hubner N, Wallace CA, Zimdahl H, Petretto E, Schulz H, Maciver F, Mueller M, Hummel O, Monti J, Zidek V, Musilova A, Kren V, Causton H, Game L, Born G, Schmidt S, Muller A, Cook SA, Kurtz TW, Whittaker J, Pravenec M, Aitman TJ (2005). Integrated transcriptional profiling and linkage analysis for identification of genes underlying disease. Nat Genet.

[B8] Bystrykh L, Weersing E, Dontje B, Sutton S, Pletcher MT, Wiltshire T, Su AI, Vellenga E, Wang J, Manly KF, Lu L, Chesler EJ, Alberts R, Jansen RC, Williams RW, Cooke MP, de Haan G (2005). Uncovering regulatory pathways that affect hematopoietic stem cell function using 'genetical genomics'. Nat Genet.

[B9] Cowles CR, Hirschhorn JN, Altshuler D, Lander ES (2002). Detection of regulatory variation in mouse genes. Nat Genet.

[B10] Schadt EE, Monks SA, Drake TA, Lusis AJ, Che N, Colinayo V, Ruff TG, Milligan SB, Lamb JR, Cavet G, Linsley PS, Mao M, Stoughton RB, Friend SH (2003). Genetics of gene expression surveyed in maize, mouse and man. Nature.

[B11] Doss S, Schadt EE, Drake TA, Lusis AJ (2005). Cis-acting expression quantitative trait loci in mice. Genome Res.

[B12] Ronald J, Brem RB, Whittle J, Kruglyak L (2005). Local Regulatory Variation in Saccharomyces cerevisiae. PLoS Genet.

[B13] Horikawa Y, Oda N, Cox NJ, Li X, Orho-Melander M, Hara M, Hinokio Y, Lindner TH, Mashima H, Schwarz PE, del Bosque-Plata L, Oda Y, Yoshiuchi I, Colilla S, Polonsky KS, Wei S, Concannon P, Iwasaki N, Schulze J, Baier LJ, Bogardus C, Groop L, Boerwinkle E, Hanis CL, Bell GI (2000). Genetic variation in the gene encoding calpain-10 is associated with type 2 diabetes mellitus. Nat Genet.

[B14] Gretarsdottir S, Thorleifsson G, Reynisdottir ST, Manolescu A, Jonsdottir S, Jonsdottir T, Gudmundsdottir T, Bjarnadottir SM, Einarsson OB, Gudjonsdottir HM, Hawkins M, Gudmundsson G, Gudmundsdottir H, Andrason H, Gudmundsdottir AS, Sigurdardottir M, Chou TT, Nahmias J, Goss S, Sveinbjornsdottir S, Valdimarsson EM, Jakobsson F, Agnarsson U, Gudnason V, Thorgeirsson G, Fingerle J, Gurney M, Gudbjartsson D, Frigge ML, Kong A, Stefansson K, Gulcher JR (2003). The gene encoding phosphodiesterase 4D confers risk of ischemic stroke. Nat Genet.

[B15] Drake TA, Schadt E, Hannani K, Kabo JM, Krass K, Colinayo V, Greaser LE, Goldin J, Lusis AJ (2001). Genetic loci determining bone density in mice with diet-induced atherosclerosis. Physiol Genomics.

[B16] Wang S, Yehya N, Schadt EE, Wang H, Drake TA, Lusis AJ (2006). Genetic and Genomic Analysis of a Fat Mass Trait with Complex Inheritance Reveals Marked Sex Specificity. PLoS Genet.

[B17] Lander ES, Botstein D (1989). Mapping mendelian factors underlying quantitative traits using RFLP linkage maps. Genetics.

[B18] Frazer KA, Wade CM, Hinds DA, Patil N, Cox DR, Daly MJ (2004). Segmental phylogenetic relationships of inbred mouse strains revealed by fine-scale analysis of sequence variation across 4.6 mb of mouse genome. Genome Res.

[B19] Cervino AC, Li G, Edwards S, Zhu J, Laurie C, Tokiwa G, Lum PY, Wang S, Castellini LW, Lusis AJ, Carlson S, Sachs AB, Schadt EE (2005). Integrating QTL and high-density SNP analyses in mice to identify Insig2 as a susceptibility gene for plasma cholesterol levels. Genomics.

[B20] Mural RJ, Adams MD, Myers EW, Smith HO, Miklos GL, Wides R, Halpern A, Li PW, Sutton GG, Nadeau J, Salzberg SL, Holt RA, Kodira CD, Lu F, Chen L, Deng Z, Evangelista CC, Gan W, Heiman TJ, Li J, Li Z, Merkulov GV, Milshina NV, Naik AK, Qi R, Shue BC, Wang A, Wang J, Wang X, Yan X, Ye J, Yooseph S, Zhao Q, Zheng L, Zhu SC, Biddick K, Bolanos R, Delcher AL, Dew IM, Fasulo D, Flanigan MJ, Huson DH, Kravitz SA, Miller JR, Mobarry CM, Reinert K, Remington KA, Zhang Q, Zheng XH, Nusskern DR, Lai Z, Lei Y, Zhong W, Yao A, Guan P, Ji RR, Gu Z, Wang ZY, Zhong F, Xiao C, Chiang CC, Yandell M, Wortman JR, Amanatides PG, Hladun SL, Pratts EC, Johnson JE, Dodson KL, Woodford KJ, Evans CA, Gropman B, Rusch DB, Venter E, Wang M, Smith TJ, Houck JT, Tompkins DE, Haynes C, Jacob D, Chin SH, Allen DR, Dahlke CE, Sanders R, Li K, Liu X, Levitsky AA, Majoros WH, Chen Q, Xia AC, Lopez JR, Donnelly MT, Newman MH, Glodek A, Kraft CL, Nodell M, Ali F, An HJ, Baldwin-Pitts D, Beeson KY, Cai S, Carnes M, Carver A, Caulk PM, Center A, Chen YH, Cheng ML, Coyne MD, Crowder M, Danaher S, Davenport LB, Desilets R, Dietz SM, Doup L, Dullaghan P, Ferriera S, Fosler CR, Gire HC, Gluecksmann A, Gocayne JD, Gray J, Hart B, Haynes J, Hoover J, Howland T, Ibegwam C, Jalali M, Johns D, Kline L, Ma DS, MacCawley S, Magoon A, Mann F, May D, McIntosh TC, Mehta S, Moy L, Moy MC, Murphy BJ, Murphy SD, Nelson KA, Nuri Z, Parker KA, Prudhomme AC, Puri VN, Qureshi H, Raley JC, Reardon MS, Regier MA, Rogers YH, Romblad DL, Schutz J, Scott JL, Scott R, Sitter CD, Smallwood M, Sprague AC, Stewart E, Strong RV, Suh E, Sylvester K, Thomas R, Tint NN, Tsonis C, Wang G, Williams MS, Williams SM, Windsor SM, Wolfe K, Wu MM, Zaveri J, Chaturvedi K, Gabrielian AE, Ke Z, Sun J, Subramanian G, Venter JC, Pfannkoch CM, Barnstead M, Stephenson LD (2002). A comparison of whole-genome shotgun-derived mouse chromosome 16 and the human genome. Science.

[B21] http://www.ncbi.nlm.nih.gov/projects/SNP/

[B22] Burke J, Davison D, Hide W (1999). d2_cluster: a validated method for clustering EST and full-length cDNAsequences. Genome Res.

[B23] Schadt EE, Edwards SW, GuhaThakurta D, Holder D, Ying L, Svetnik V, Leonardson A, Hart KW, Russell A, Li G, Cavet G, Castle J, McDonagh P, Kan Z, Chen R, Kasarskis A, Margarint M, Caceres RM, Johnson JM, Armour CD, Garrett-Engele PW, Tsinoremas NF, Shoemaker DD (2004). A comprehensive transcript index of the human genome generated using microarrays and computational approaches. Genome Biol.

[B24] Cawley S, Bekiranov S, Ng HH, Kapranov P, Sekinger EA, Kampa D, Piccolboni A, Sementchenko V, Cheng J, Williams AJ, Wheeler R, Wong B, Drenkow J, Yamanaka M, Patel S, Brubaker S, Tammana H, Helt G, Struhl K, Gingeras TR (2004). Unbiased mapping of transcription factor binding sites along human chromosomes 21 and 22 points to widespread regulation of noncoding RNAs. Cell.

[B25] Loots GG, Locksley RM, Blankespoor CM, Wang ZE, Miller W, Rubin EM, Frazer KA (2000). Identification of a coordinate regulator of interleukins 4, 13, and 5 by cross-species sequence comparisons. Science.

[B26] Wasserman WW, Palumbo M, Thompson W, Fickett JW, Lawrence CE (2000). Human-mouse genome comparisons to locate regulatory sites. Nat Genet.

[B27] Elnitski L, Hardison RC, Li J, Yang S, Kolbe D, Eswara P, O'Connor MJ, Schwartz S, Miller W, Chiaromonte F (2003). Distinguishing regulatory DNA from neutral sites. Genome Res.

[B28] Lenhard B, Sandelin A, Mendoza L, Engstrom P, Jareborg N, Wasserman WW (2003). Identification of conserved regulatory elements by comparative genome analysis. J Biol.

[B29] Cooper GM, Sidow A (2003). Genomic regulatory regions: insights from comparative sequence analysis. Curr Opin Genet Dev.

[B30] Levy S, Hennenhalli S, Workman C (2001). Enrichment of regulatory signals in conserved non-coding genomic sequence.. Bioinformatics.

[B31] Karolchik D, Baertsch R, Diekhans M, Furey TS, Hinrichs A, Lu YT, Roskin KM, Schwartz M, Sugnet CW, Thomas DJ, Weber RJ, Haussler D, Kent WJ (2003). The UCSC Genome Browser Database. Nucleic Acids Res.

[B32] Schwartz S, Kent WJ, Smit A, Zhang Z, Baertsch R, Hardison RC, Haussler D, Miller W (2003). Human-mouse alignments with BLASTZ. Genome Res.

[B33] Waterston RH, Lindblad-Toh K, Birney E, Rogers J, Abril JF, Agarwal P, Agarwala R, Ainscough R, Alexandersson M, An P, Antonarakis SE, Attwood J, Baertsch R, Bailey J, Barlow K, Beck S, Berry E, Birren B, Bloom T, Bork P, Botcherby M, Bray N, Brent MR, Brown DG, Brown SD, Bult C, Burton J, Butler J, Campbell RD, Carninci P, Cawley S, Chiaromonte F, Chinwalla AT, Church DM, Clamp M, Clee C, Collins FS, Cook LL, Copley RR, Coulson A, Couronne O, Cuff J, Curwen V, Cutts T, Daly M, David R, Davies J, Delehaunty KD, Deri J, Dermitzakis ET, Dewey C, Dickens NJ, Diekhans M, Dodge S, Dubchak I, Dunn DM, Eddy SR, Elnitski L, Emes RD, Eswara P, Eyras E, Felsenfeld A, Fewell GA, Flicek P, Foley K, Frankel WN, Fulton LA, Fulton RS, Furey TS, Gage D, Gibbs RA, Glusman G, Gnerre S, Goldman N, Goodstadt L, Grafham D, Graves TA, Green ED, Gregory S, Guigo R, Guyer M, Hardison RC, Haussler D, Hayashizaki Y, Hillier LW, Hinrichs A, Hlavina W, Holzer T, Hsu F, Hua A, Hubbard T, Hunt A, Jackson I, Jaffe DB, Johnson LS, Jones M, Jones TA, Joy A, Kamal M, Karlsson EK, Karolchik D, Kasprzyk A, Kawai J, Keibler E, Kells C, Kent WJ, Kirby A, Kolbe DL, Korf I, Kucherlapati RS, Kulbokas EJ, Kulp D, Landers T, Leger JP, Leonard S, Letunic I, Levine R, Li J, Li M, Lloyd C, Lucas S, Ma B, Maglott DR, Mardis ER, Matthews L, Mauceli E, Mayer JH, McCarthy M, McCombie WR, McLaren S, McLay K, McPherson JD, Meldrim J, Meredith B, Mesirov JP, Miller W, Miner TL, Mongin E, Montgomery KT, Morgan M, Mott R, Mullikin JC, Muzny DM, Nash WE, Nelson JO, Nhan MN, Nicol R, Ning Z, Nusbaum C, O'Connor MJ, Okazaki Y, Oliver K, Overton-Larty E, Pachter L, Parra G, Pepin KH, Peterson J, Pevzner P, Plumb R, Pohl CS, Poliakov A, Ponce TC, Ponting CP, Potter S, Quail M, Reymond A, Roe BA, Roskin KM, Rubin EM, Rust AG, Santos R, Sapojnikov V, Schultz B, Schultz J, Schwartz MS, Schwartz S, Scott C, Seaman S, Searle S, Sharpe T, Sheridan A, Shownkeen R, Sims S, Singer JB, Slater G, Smit A, Smith DR, Spencer B, Stabenau A, Stange-Thomann N, Sugnet C, Suyama M, Tesler G, Thompson J, Torrents D, Trevaskis E, Tromp J, Ucla C, Ureta-Vidal A, Vinson JP, Von Niederhausern AC, Wade CM, Wall M, Weber RJ, Weiss RB, Wendl MC, West AP, Wetterstrand K, Wheeler R, Whelan S, Wierzbowski J, Willey D, Williams S, Wilson RK, Winter E, Worley KC, Wyman D, Yang S, Yang SP, Zdobnov EM, Zody MC, Lander ES (2002). Initial sequencing and comparative analysis of the mouse genome. Nature.

[B34] Matys V, Fricke E, Geffers R, Gossling E, Haubrock M, Hehl R, Hornischer K, Karas D, Kel AE, Kel-Margoulis OV, Kloos DU, Land S, Lewicki-Potapov B, Michael H, Munch R, Reuter I, Rotert S, Saxel H, Scheer M, Thiele S, Wingender E (2003). TRANSFAC: transcriptional regulation, from patterns to profiles. Nucleic Acids Res.

[B35] Stormo GD, Fields DS (1998). Specificity, free energy and information content in protein-DNA interactions. Trends Biochem Sci.

[B36] Stormo GD (2000). DNA binding sites: representation and discovery. Bioinformatics.

[B37] Benos PV, Bulyk ML, Stormo GD (2002). Additivity in protein-DNA interactions: how good an approximation is it?. Nucleic Acids Res.

[B38] GuhaThakurta D (2006). Computational identification of transcriptional regulatory elements in DNA sequence. Nucleic Acids Res.

[B39] Conde L, Vaquerizas JM, Santoyo J, Al-Shahrour F, Ruiz-Llorente S, Robledo M, Dopazo J (2004). PupaSNP Finder: a web tool for finding SNPs with putative effect at transcriptional level. Nucleic Acids Res.

[B40] Mottagui-Tabar S, Faghihi MA, Mizuno Y, Engstrom PG, Lenhard B, Wasserman WW, Wahlestedt C (2005). Identification of functional SNPs in the 5-prime flanking sequences of human genes. BMC Genomics.

[B41] Zhao T, Chang LW, McLeod HL, Stormo GD (2004). PromoLign: a database for upstream region analysis and SNPs. Hum Mutat.

[B42] Ponomarenko JV, Orlova GV, Merkulova TI, Gorshkova EV, Fokin ON, Vasiliev GV, Frolov AS, Ponomarenko MP (2002). rSNP_Guide: an integrated database-tools system for studying SNPs and site-directed mutations in transcription factor binding sites. Hum Mutat.

[B43] Kel AE, Gossling E, Reuter I, Cheremushkin E, Kel-Margoulis OV, Wingender E (2003). MATCH: A tool for searching transcription factor binding sites in DNA sequences. Nucleic Acids Res.

[B44] Wasserman WW, Sandelin A (2004). Applied bioinformatics for the identification of regulatory elements. Nat Rev Genet.

[B45] Bulyk ML (2003). Computational prediction of transcription-factor binding site locations. Genome Biol.

[B46] Kim SK, Lund J, Kiraly M, Duke K, Jiang M, Stuart JM, Eizinger A, Wylie BN, Davidson GS (2001). A gene expression map for Caenorhabditis elegans. Science.

[B47] Zhu Z, Pilpel Y, Church GM (2002). Computational identification of transcription factor binding sites via a transcription-factor-centric clustering (TFCC) algorithm. J Mol Biol.

[B48] Stuart JM, Segal E, Koller D, Kim SK (2003). A gene-coexpression network for global discovery of conserved genetic modules. Science.

[B49] Shoemaker DD, Schadt EE, Armour CD, He YD, Garrett-Engele P, McDonagh PD, Loerch PM, Leonardson A, Lum PY, Cavet G, Wu LF, Altschuler SJ, Edwards S, King J, Tsang JS, Schimmack G, Schelter JM, Koch J, Ziman M, Marton MJ, Li B, Cundiff P, Ward T, Castle J, Krolewski M, Meyer MR, Mao M, Burchard J, Kidd MJ, Dai H, Phillips JW, Linsley PS, Stoughton R, Scherer S, Boguski MS (2001). Experimental annotation of the human genome using microarray technology. Nature.

[B50] Castle J, Garrett-Engele P, Armour CD, Duenwald SJ, Loerch PM, Meyer MR, Schadt EE, Stoughton R, Parrish ML, Shoemaker DD, Johnson JM (2003). Optimization of oligonucleotide arrays and RNA amplification protocols for analysis of transcript structure and alternative splicing. Genome Biol.

[B51] Hoogendoorn B, Coleman SL, Guy CA, Smith K, Bowen T, Buckland PR, O'Donovan MC (2003). Functional analysis of human promoter polymorphisms. Hum Mol Genet.

[B52] Firulli AB (2003). A HANDful of questions: the molecular biology of the heart and neural crest derivatives (HAND)-subclass of basic helix-loop-helix transcription factors. Gene.

[B53] Pastinen T, Sladek R, Gurd S, Sammak A, Ge B, Lepage P, Lavergne K, Villeneuve A, Gaudin T, Brandstrom H, Beck A, Verner A, Kingsley J, Harmsen E, Labuda D, Morgan K, Vohl MC, Naumova AK, Sinnett D, Hudson TJ (2004). A survey of genetic and epigenetic variation affecting human gene expression. Physiol Genomics.

[B54] Liao G, Wang J, Guo J, Allard J, Cheng J, Ng A, Shafer S, Puech A, McPherson JD, Foernzler D, Peltz G, Usuka J (2004). In silico genetics: identification of a functional element regulating H2-Ealpha gene expression. Science.

[B55] Mooney S (2005). Bioinformatics approaches and resources for single nucleotide polymorphism functional analysis. Brief Bioinform.

[B56] Rockman MV, Wray GA (2002). Abundant raw material for cis-regulatory evolution in humans. Mol Biol Evol.

[B57] Buckland PR, Hoogendoorn B, Guy CA, Coleman SL, Smith SK, Buxbaum JD, Haroutunian V, O'Donovan MC (2004). A high proportion of polymorphisms in the promoters of brain expressed genes influences transcriptional activity. Biochim Biophys Acta.

[B58] Buckland PR, Hoogendoorn B, Coleman SL, Guy CA, Smith SK, O'Donovan MC (2005). Strong bias in the location of functional promoter polymorphisms. Hum Mutat.

[B59] Buckland PR, Coleman SL, Hoogendoorn B, Guy C, Smith SK, O'Donovan MC (2004). A high proportion of chromosome 21 promoter polymorphisms influence transcriptional activity. Gene Expr.

[B60] Pastinen T, Ge B, Gurd S, Gaudin T, Dore C, Lemire M, Lepage P, Harmsen E, Hudson TJ (2005). Mapping common regulatory variants to human haplotypes. Hum Mol Genet.

[B61] Lee PD, Ge B, Greenwood CM, Sinnett D, Fortin Y, Brunet S, Fortin A, Takane M, Skamene E, Pastinen T, Hallett M, Hudson TJ, Sladek R (2006). Mapping cis-acting regulatory variation in recombinant congenic strains. Physiol Genomics.

[B62] Yuh CH, Bolouri H, Davidson EH (1998). Genomic cis-regulatory logic: experimental and computational analysis of a sea urchin gene. Science.

[B63] r_SNP guide examples. http://wwwmgs.bionet.nsc.ru/mgs/programs/rsnp/images/.

[B64] Stenson PD, Ball EV, Mort M, Phillips AD, Shiel JA, Thomas NS, Abeysinghe S, Krawczak M, Cooper DN (2003). Human Gene Mutation Database (HGMD): 2003 update. Hum Mutat.

[B65] Herbert A, Gerry NP, McQueen MB, Heid IM, Pfeufer A, Illig T, Wichmann HE, Meitinger T, Hunter D, Hu FB, Colditz G, Hinney A, Hebebrand J, Koberwitz K, Zhu X, Cooper R, Ardlie K, Lyon H, Hirschhorn JN, Laird NM, Lenburg ME, Lange C, Christman MF (2006). A common genetic variant is associated with adult and childhood obesity. Science.

[B66] Florez JC, Jablonski KA, Bayley N, Pollin TI, de Bakker PI, Shuldiner AR, Knowler WC, Nathan DM, Altshuler D (2006). TCF7L2 polymorphisms and progression to diabetes in the Diabetes Prevention Program. N Engl J Med.

[B67] Harbison CT, Gordon DB, Lee TI, Rinaldi NJ, Macisaac KD, Danford TW, Hannett NM, Tagne JB, Reynolds DB, Yoo J, Jennings EG, Zeitlinger J, Pokholok DK, Kellis M, Rolfe PA, Takusagawa KT, Lander ES, Gifford DK, Fraenkel E, Young RA (2004). Transcriptional regulatory code of a eukaryotic genome. Nature.

[B68] Mehrabian M, Allayee H, Stockton J, Lum PY, Drake TA, Castellani LW, Suh M, Armour C, Edwards S, Lamb J, Lusis AJ, Schadt EE (2005). Integrating genotypic and expression data in a segregating mouse population to identify 5-lipoxygenase as a susceptibility gene for obesity and bone traits. Nat Genet.

[B69] UCSC mouse-human alignments. http://genome-archive.cse.ucsc.edu/goldenPath/mm4/vsHg16/.

[B70] Hardenbol P, Yu F, Belmont J, Mackenzie J, Bruckner C, Brundage T, Boudreau A, Chow S, Eberle J, Erbilgin A, Falkowski M, Fitzgerald R, Ghose S, Iartchouk O, Jain M, Karlin-Neumann G, Lu X, Miao X, Moore B, Moorhead M, Namsaraev E, Pasternak S, Prakash E, Tran K, Wang Z, Jones HB, Davis RW, Willis TD, Gibbs RA (2005). Highly multiplexed molecular inversion probe genotyping: over 10,000 targeted SNPs genotyped in a single tube assay. Genome Res.

[B71] He YD, Dai H, Schadt EE, Cavet G, Edwards SW, Stepaniants SB, Duenwald S, Kleinhanz R, Jones AR, Shoemaker DD, Stoughton RB (2003). Microarray standard data set and figures of merit for comparing data processing methods and experiment designs. Bioinformatics.

[B72] QTL Cartographer. http://statgen.ncsu.edu/qtlcart/.

[B73] Conti E, Izaurralde E (2005). Nonsense-mediated mRNA decay: molecular insights and mechanistic variations across species. Curr Opin Cell Biol.

[B74] Bailey JA, Church DM, Ventura M, Rocchi M, Eichler EE (2004). Analysis of segmental duplications and genome assembly in the mouse. Genome Res.

[B75] Map of genomic duplications in mouse. http://mouseparalogy.gs.washington.edu/.

[B76] Pletcher MT, McClurg P, Batalov S, Su AI, Barnes SW, Lagler E, Korstanje R, Wang X, Nusskern D, Bogue MA, Mural RJ, Paigen B, Wiltshire T (2004). Use of a dense single nucleotide polymorphism map for in silico mapping in the mouse. PLoS Biol.

